# Supplementary material for: Semi-Volatile Organic Compounds in Car Dust: A Pilot Study in Jeddah, Saudi Arabia
Source: Int J Environ Res Public Health. 2021 Apr 30;18(9):4803. doi: 10.3390/ijerph18094803 (PMC8124401; doi:10.3390/ijerph18094803)
Supplement: Supplementary file 1 [file ijerph-18-04803-s001.zip › ijerph-1188448-supplementary.pdf]

International Journal of *Environmental Research and Public Health*

MDPI

Supplementary material

Semivolatile Organic Compounds in Car Dust: A Pilot Study in Jeddah, Saudi Arabia

Nadeem Ali [1,*], Mohammad W. Kadi [2], Hussain Mohammed Salem Ali Albar [3], Muhammad Imtiaz Rashid [1], Sivaraman Chandrasekaran [1], Ahmed Saleh Summan [1,4], Cynthia A. de Wit [5] and Govindan Malarvannan [6,*]

[1] Centre of Excellence in Environmental Studies, King Abdulaziz University, Jeddah 21589, Saudi Arabia; mimurad@kau.edu.sa (M.I.R.); scsekaran@kau.edu.sa (S.C.); asumman@kau.edu.sa (A.S.S.)
[2] Department of Chemistry, Faculty of Sciences, King Abdul Aziz University, Jeddah 21589, Saudi Arabia; mkadi@kau.edu.sa
[3] Department of Community Health, Medical College, King Abdul Aziz University, Jeddah 21589, Saudi Arabia; hmalbar@kau.edu.sa
[4] Department of Environmental Sciences, King Abdul Aziz University, Jeddah 21589, Saudi Arabia
[5] Department of Environment Science, Stockholm University, 11419 Stockholm, Sweden; Cynthia.deWit@aces.su.se
[6] Toxicological Center, University of Antwerp, 2610 Antwerpen, Belgium
* Correspondence: nabahadar@kau.edu.sa (N.A.); malarvannan.govindan@uantwerpen.be (G.M.)

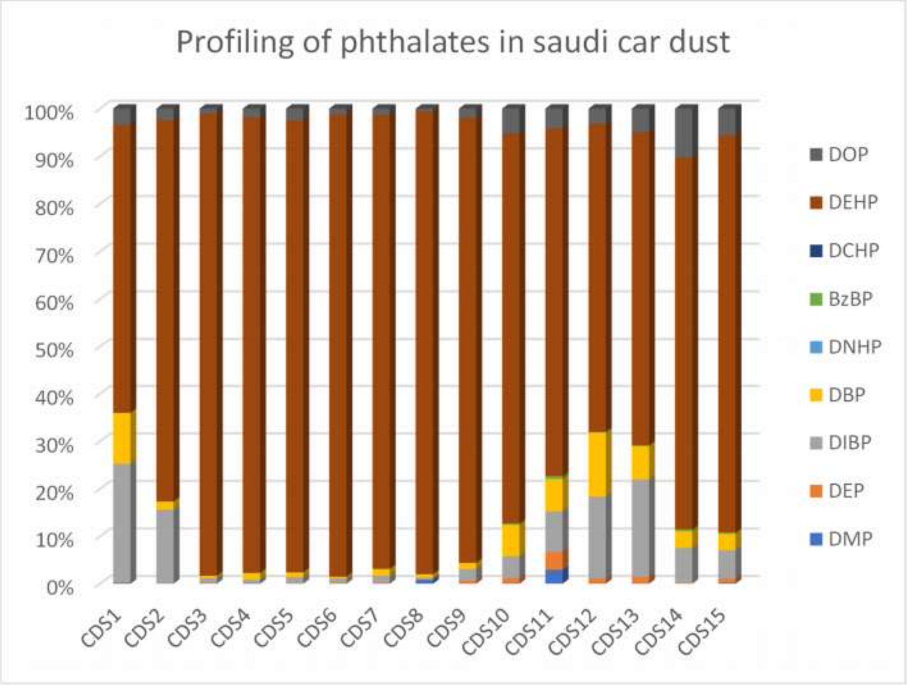

Figure S1. Profile of phthalates in each car dust sample.

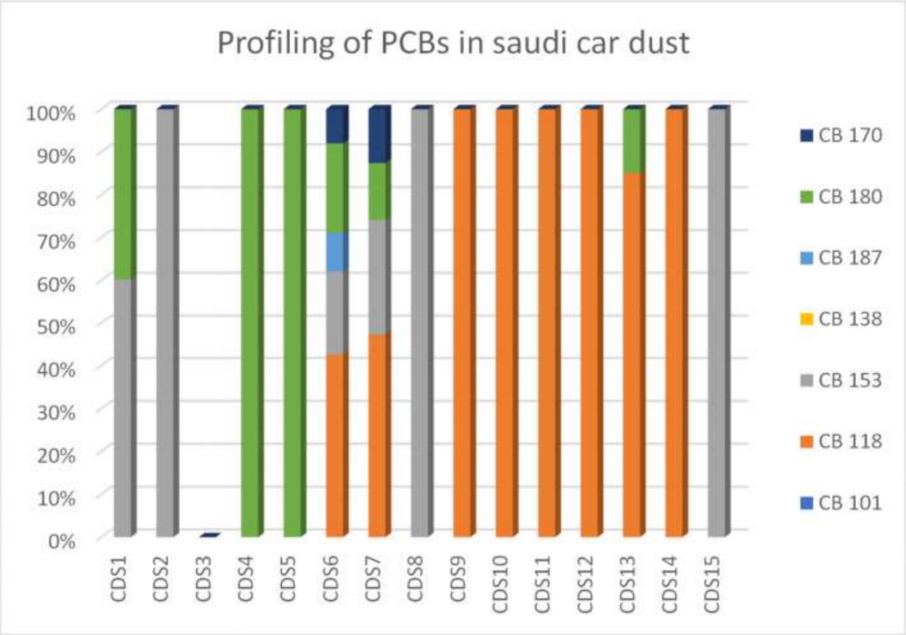

Figure S2. Profile of PCB congeners in each car dust sample.

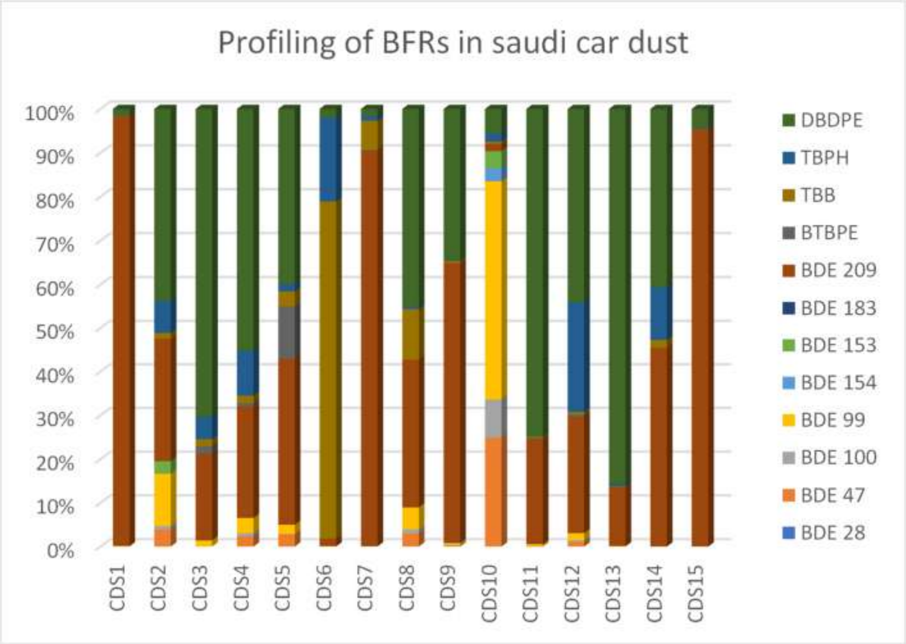

Figure S3. Profile of BFRs in each car dust sample.

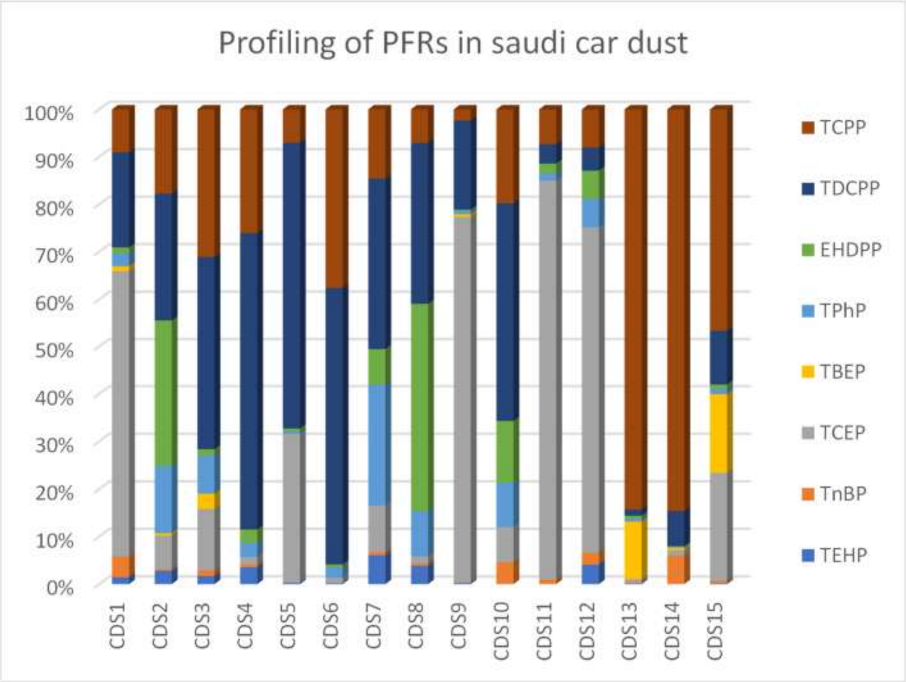

Figure S4. Profile of OPFRs in each car dust sample.

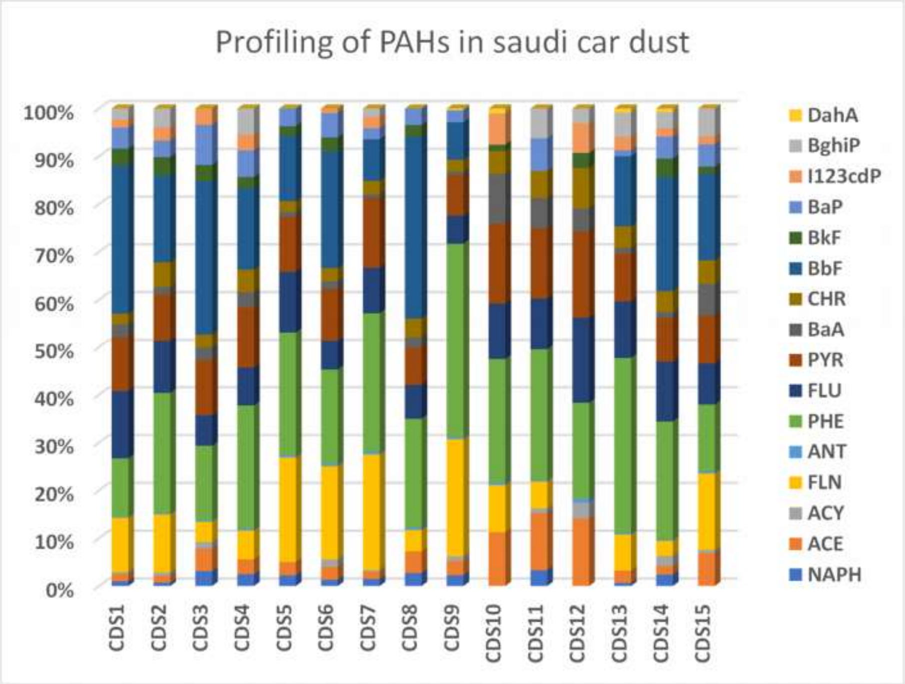

Figure S5. Profile of PAHs in each car dust sample.

Table S1. The estimated daily exposure (mean, median, 25th percentile, maximum) of taxi and regular drivers *via* car dust compared to reference dose values (RfD) from the literature. All values are given in ng/kg BW/day.

		Regular Driver				Taxi Driver			
	**RfD**	**Mean**	**Median**	**25th P**	**Max**	**Mean**	**Median**	**25th P**	**Max**
NAPH		<0.1	<0.1	<0.1	<0.1	0.1	0.1	<0.1	0.2
ACE	60[1]	<0.1	<0.1	<0.1	<0.1	0.1	0.1	0.1	0.2
ACY		<0.1	<0.1	<0.1	<0.1	<0.1	<0.1	<0.1	0.1
FLN	40[1]	0.1	<0.1	<0.1	0.3	0.6	0.3	<0.1	1.6
ANT		<0.1	<0.1	<0.1	<0.1	<0.1	<0.1	<0.1	<0.1
PHE	40[1]	0.2	0.2	<0.1	0.4	1.1	0.8	0.2	2.2
FLU		0.1	0.1	<0.1	0.2	0.5	0.3	0.1	1.2
PYR	30[1]	0.1	0.1	<0.1	0.2	0.5	0.4	0.1	1.2
BaA		<0.1	<0.1	<0.1	<0.1	0.1	0.1	<0.1	0.2
CHR		<0.1	<0.1	<0.1	0.1	0.2	0.1	<0.1	0.3
BbF		0.2	0.1	<0.1	0.5	0.8	0.7	<0.1	2.3
BkF		<0.1	<0.1	<0.1	0.1	0.1	<0.1	<0.1	0.3
BaP	0.14*[1]	<0.1	<0.1	<0.1	0.1	0.2	0.2	<0.1	0.4
I123cdP		<0.1	<0.1	<0.1	<0.1	0.1	<0.1	<0.1	0.2
BghiP		<0.1	<0.1	<0.1	0.1	0.1	0.1	<0.1	0.3
DahA		<0.1	<0.1	<0.1	<0.1	<0.1	<0.1	<0.1	<0.1
**ΣPAHs**		**0.9**	**0.7**	**0.1**	**1.8**	**4.4**	**3.5**	**0.7**	**9.1**
DMP		0.4	0.1	<0.1	1.8	2.1	0.6	0.2	8.9
DEP	800000[2]	0.6	0.4	0.2	1.9	3.1	1.9	1.2	9.6
DIBP		28.3	4.5	1.3	129.8	142.8	22.7	6.6	654.5
DBP	100000[2]	11.1	4.6	1.3	43.7	55.9	23.3	6.4	220.1
DNHP		<0.1	<0.1	<0.1	<0.1	<0.1	<0.1	<0.1	<0.1
BzBP	200000[2]	0.4	0.1	0.1	1.3	2.0	0.8	0.3	6.4
DCHP		<0.1	<0.1	<0.1	<0.1	<0.1	<0.1	<0.1	<0.1
DEHP	20000[2]	278.7	296.4	22.2	538.8	1405.3	1494.7	111.9	2716.7
DOP		11.4	4.5	1.3	41.6	57.4	22.8	6.4	209.5
**Σ Phthalates**		**330.9**	**304.5**	**27.7**	**758.1**	**1668.6**	**1535.3**	**139.8**	**3822.3**
TEHP		<0.1	<0.1	<0.1	0.1	0.2	0.2	<0.1	0.7
TnBP	24000[3]	0.1	<0.1	<0.1	0.5	0.6	0.1	<0.1	2.7
TCEP	22000[3]	1.7	0.3	<0.1	6.7	8.4	1.5	0.1	34.0
TBEP	15000[3]	0.4	<0.1	<0.1	2.6	1.9	<0.1	<0.1	12.9
TPhP	70000[3]	0.2	0.1	<0.1	0.6	0.9	0.6	0.1	3.1
EHDPP		0.2	<0.1	<0.1	1.1	1.2	0.2	0.1	5.6
TDCPP	15000[3]	2.1	0.6	<0.1	9.1	10.6	3.2	0.2	45.7
TCPP	80000[3]	3.9	0.4	<0.1	18.2	19.5	2.0	0.2	91.7
**ΣOPFRs**		**8.6**	**2.5**	**0.4**	**21.5**	**43.5**	**12.6**	**1.8**	**108.6**
BDE 28		<0.1	<0.1	<0.1	<0.1	<0.1	<0.1	<0.1	<0.1
BDE 47	100[4]	<0.1	<0.1	<0.1	<0.1	<0.1	<0.1	<0.1	0.1
BDE 100		<0.1	<0.1	<0.1	<0.1	<0.1	<0.1	<0.1	<0.1
BDE 99	100[4]	<0.1	<0.1	<0.1	<0.1	<0.1	<0.1	<0.1	0.2
BDE 154		<0.1	<0.1	<0.1	<0.1	<0.1	<0.1	<0.1	<0.1
BDE 153	200[4]	<0.1	<0.1	<0.1	<0.1	<0.1	<0.1	<0.1	<0.1
BDE 183		<0.1	<0.1	<0.1	<0.1	<0.1	<0.1	<0.1	<0.1
BDE 209	7000[4]	1.4	<0.1	<0.1	6.9	7.1	0.2	<0.1	35.0
BTBPE	243000[5]	<0.1	<0.1	<0.1	<0.1	<0.1	<0.1	<0.1	0.1
TBB	20000[5]	0.2	<0.1	<0.1	1.1	0.9	<0.1	<0.1	5.3
TBPH	20000[5]	0.1	<0.1	<0.1	0.2	0.3	<0.1	<0.1	1.2
DBDPE	333333[5]	0.2	0.1	<0.1	0.7	1.0	0.3	0.1	3.5
**ΣBFRs**		**1.9**	**0.2**	**0.1**	**7.4**	**9.3**	**1.0**	**0.4**	**37.4**
CB 101		<0.1	<0.1	<0.1	<0.1	<0.1	<0.1	<0.1	<0.1
CB 118		<0.1	<0.1	<0.1	<0.1	<0.1	<0.1	<0.1	<0.1
CB 153		<0.1	<0.1	<0.1	<0.1	<0.1	<0.1	<0.1	<0.1
CB 138		<0.1	<0.1	<0.1	<0.1	<0.1	<0.1	<0.1	<0.1
CB 187		<0.1	<0.1	<0.1	<0.1	<0.1	<0.1	<0.1	<0.1
CB 180		<0.1	<0.1	<0.1	<0.1	<0.1	<0.1	<0.1	<0.1
CB 170		<0.1	<0.1	<0.1	<0.1	<0.1	<0.1	<0.1	<0.1
**ΣPCBs**	**30[6]**	**<0.1**	**<0.1**	**<0.1**	**<0.1**	**<0.1**	**<0.1**	**<0.1**	**<0.1**

**ΣAll chemicals**	**342.3**	**306.5**	**28.6**	**780.3**	**1725.9**	**1545.5**	**144.0**	**3934.3**

*Virtually safe dose; [1]Bulder et al., 2006; [2]ATSDR, 2001; [3]Ali et al., 2012; [4]EPA, 2008; [5]Hardy et al., 2008; [6]ATSDR, 2000.

References

1. Bulder, A.S., Hoogenboom, L.A.P., Kan, C.A., Van Raamsdonk, L.W.D., Traag, W.A. and Bouwmeester, H., 2006. Initial risk assessment of polycyclic aromatic hydrocarbons (PAHs) in feed (materials) (No. 2006.001). RIKILT.
2. ATSDR, 2001. Toxicological profile for di-n-butyl phthalate. U.S. Department of Health and Human Services, Public Health Service, Agency for Toxic Substances and Disease Registry, September 2001, U.S. Department of Health and Human Services.
3. Ali, N., Van den Eede, N., Dirtu, A.C., Neels, H., Covaci, A., 2012b. Assessment of human exposure to indoor organic contaminants via dust ingestion in Pakistan. Indoor Air 22, 200–211.
4. EPA (USA Environmental Protection Agency), 2008. Integrated risk information system, http://www.epa.gov/iris/subst/0294.htm (accessed on 05/10/2012).
5. Hardy, M., Biesemeir, J., Banasik, M., Stedeford, T., 2008. Brominated flame retardants in house dust: Detection does not equate to risk. Environ. Sci. Technol. 42, 9453-9454.
6. ATSDR (Agency for Toxic Substances and Disease Registry), 2000. Toxicological profile for polychlorinated biphenyls (PCBs), Atlanta.
